# An Optimized Artificial Intelligence System Using IoT Biosensors Networking for Healthcare Problems

**DOI:** 10.1155/2022/2206573

**Published:** 2022-03-24

**Authors:** Shadab Khan, Yash Veer Singh, Pushpendra Singh, Ram Sewak Singh

**Affiliations:** ^1^Department of Computer Science & Engineering, Sunder Deep Engineering College, Ghaziabad, UP 201002, India; ^2^Department of Information Technology, ABES Engineering College, Ghaziabad, UP 201009, India; ^3^Department of Information Technology, Raj Kumar Goel Institute of Technology, Meerut Road, Ghaziabad, UP 101003, India; ^4^Department of Electronics and Communication, School of Electrical Engineering and Computing, Adama Science and Technology University, Adama, Ethiopia

## Abstract

In today's environment, electronics technology is growing rapidly because of the availability of the numerous and latest devices which can be deployed for monitoring and controlling the various healthcare systems. Due to the limitations of such devices, there is a dire need to optimize the utilization of the devices. In healthcare systems, Internet of things (IoT) based biosensors networking has minimal energy during transmission and collecting data. This paper proposes an optimized artificial intelligence system using IoT biosensors networking for healthcare problems for efficient data collection from the deployed sensor nodes. Here, an optimized tunicate swarm algorithm is used for optimizing the route for data collection and transmission among the patient and doctor. The fitness function of the optimized tunicate swarm algorithm used the distance, proximity, residual, and average energy of nodes parameters. The proposed method is attributed to the optimal CH chosen under TSA operation having a lower energy consumption. The performance of the proposed method is compared to the existing methods in terms of various metrics like stability period, lifetime, throughput, and clusters per round.

## 1. Introduction

Nowadays, technologies are developing rapidly due to their enhancement in electronics devices, especially in their cost and effectiveness of performance. These technologies have many applications that provide many effective solutions from healthcare to agriculture and military to industry. The innovative solutions are significantly implemented in healthcare organizations, enhancing the treatment and its performance. These innovative solutions transform the industrial revolution significantly. In the healthcare systems, these technologies provide a complete and new paradigm for the treatment of the patients where patients' health can be monitored entirely, and management of patient health can be done on a real-time basis in an efficient and effective manner [1]. There are various areas in the healthcare system where the Internet of things (IoTs) based systems can be implemented such as web health portals mobile applications, biosensors, blockchain-based electronic medical record systems, smart devices, home virtual assistants, wearables, predictive analytics, etc. When we are looking for improving the existing healthcare systems then only one thing that came into mind is digital healthcare. In digital healthcare, the existing infrastructure can be improved by improving the automated diagnoses decisions, data analysis in an intelligent way, enhancing the treatment process, continuous patient state monitoring, and healthier existing customer services [2].

As a result of technological breakthroughs in the disciplines of microelectromechanical systems (MEMS) and wireless communication systems, IoT-enabled wireless sensor networks have emerged as one of the fastest-growing sectors in the world. IoT-enabled WSNs are made up of many tiny battery-operated devices known as sensor devices that communicate with one another. They are equipped with three fundamental principles: (1) data sensing from the external environment, (2) processing on the detected data, and (3) data transfer through radio frequency (RF). The IoT-enabled WSNs are comprised of sensors that are capable of providing expert solutions in contexts where human involvement is complicated, such as military surveillance, structure health monitoring, natural catastrophe forecasting, and traffic management, among others. Following their installation in the IoT-enabled WSNs, these sensor devices collect data from their surroundings, perform computer processing on the data they receive, and then communicate the data to the BS for further processing [3]. Being compact in size, sensor devices have restricted power backup, storage capacities, and computing potentials due to their small size. Once deployed in the network field, these sensor devices are left unattended, giving rise to the fundamental problem of ensuring that each sensor device's energy is utilized as efficiently as possible.

As a result, the development of energy-efficient protocols can aid in the efficient usage of energy by each SN, hence ensuring the network's long-term viability. The organization of these sensor devices into tiny groups to create clusters is critical in the construction of such efficient protocols, and clustering plays an essential part in this. Clustered networks are used in the clustered network strategy, in which a WSN is partitioned into smaller groups. Each cluster is made up of a small number of sensor devices and a cluster head (CH), who serves as a leader. All sensor devices transfer the data they have collected to their respective CHs, which in turn relay the data to the BS. As a result, effective CH selection, the optimal number of clusters, cluster maintenance, and data routing to the BS are all essential considerations in the creation of clustering-based energy-efficient protocols, among other things [4]. In order to address the concerns raised above, several clustering procedures have been developed by a variety of writers. However, identifying these clustering concerns is only one aspect of the problem; sustaining quality of service (QoS) and balancing trade-offs between contradicting requirements such as lifespan, coverage, and throughput need also be addressed professionally. Recently, bioinspired or metaheuristic approaches and expert systems have received a great deal of interest for their ability to deal with these difficulties [5, 6].

Various energy-efficient protocols have been developed in recent years for either homogeneous or heterogeneous networks, depending on the application. According to conventional wisdom, homogeneous networks are made up of sensor devices that have the same amount of energy resources at the start of the network. In contrast, heterogeneous networks have sensor devices with varying amounts of energy resources. Initially, a homogeneous model is a specific type of WSN. Each sensor device has the same energy resources but eventually becomes a heterogeneous model as the network operates. Each SN cannot waste the same energy resource due to differences in radio communication characteristics. The incidence of random events or the geometrical factors of the network field is a result of these differences. Notably, it demonstrates that developing an energy-efficient protocol capable of functioning in both homogeneous and heterogeneous networks is a significant issue [7–9]. In this study, we offer a method that considers both homogeneous and heterogeneous network models.

The rest of the paper is structured as follows: In Section 2, the literature review of the existing techniques is discussed. Sections 3 and 4 discuss the system model and proposed methodology, respectively. The simulation results and discussion are given in Section 5 and finally, the paper is concluded in Section 6.

## 2. Literature Review

In this section, a review of the various existing techniques is given as follows: The clustering technique has been shown to be a significant component in the design of energy-efficient protocols in IoT-enabled wireless sensor networks (WSNs). Many clustering-based protocols have been developed over the last two decades, including LEACH [10], LEACH-C [11], LEACH-M [12], HEED [13], and others. Furthermore, LEACH is used as a benchmark and as the ascendant protocol for the majority of protocols in this industry. LEACH is a self-organizing, distributed clustering technology that assumes that each SN in the WSN consumes the same amount of energy. The LEACH working module is separated into the setup phase and the steady-state phase. As part of the setup phase, each SN conducts a CH election operation that is guided by a probabilistic methodology. Once clusters are created in a steady phase, each SN senses its surroundings in order to gather data, which is then forwarded to the corresponding CH. Each CH combines the data received from its cluster members and delivers it to the BS, which is located thousands of miles distant, through a direct connection. LEACH-C is a centralized variation of LEACH, in which the BS is in charge of the CH selection process rather than the CH. At the start of each round, all sensor devices transmit their current position information and their current energy level to the BS. Accordingly, the network's average energy is calculated. Only those sensor devices can participate in the CH selection process with values more significant than the average energy for the current round. Once the BS has chosen the CHs, it broadcasts the positions of the CHs in the network, and all other sensor devices join the CH that is closest to them. Because of the centralized method, LEACH-C, on the other hand, suffers from scalability difficulties.

LEACH-M is a variation on the LEACH technique that includes multiple hops. Between CHs and BSs, writers compared the performance of a multihop communication scheme and a single-hop communication strategy using this method to see which performed better. The results clearly demonstrated that LEACH-M outperformed LEACH in terms of performance. HEED, which is one of the most well-known protocols in this field, is a hybrid, distributed, and iteration-based clustering protocol that uses a hybrid approach. According to this technique, the selection of the CH is determined by using a hybrid combination of residual energy and intracluster communication cost. First and foremost, HEED is hampered by the creation of a large number of clusters. Second, owing to the random selection of CHs at the beginning of the process, there is a great deal of variance in the number of CHs in each successive round. Huang and Wu [14] provide an expansion on the HEED concept.

In HEED, sensor devices that were left unnoticed by the CHs and were compelled to elect themselves as CHs resulted in the creation of extra CHs in the system. This difficulty was solved by employing a re-election process on uncovered sensor devices, which increased the efficiency of the HEED algorithm. MiCRA [15] is another innovation in HEED that employs a two-level hierarchy on the approach for selecting the best candidate. At the first level, CHs are selected in the same way as HEED; however, at the second level, only those CHs who were elected at the first level are eligible to participate in the CHs selection procedure. In the network, this sort of hierarchical cluster creation aids in distributing energy among the nodes (sensor devices). Sabet and Naji [16] suggested a distributed clustering strategy using multihop routing algorithms that may efficiently limit the energy consumption caused by control packets while yet maintaining high performance. Du et al. [17] presented an EESSC protocol that employed the residual energy parameter for clustering methods and used a specific packet header to update the sensor devices' residual energy during data transmission in the network while data transmission was taking place. Gupta and Sharma [18] suggested an ICHB method for the CH selection procedure that was based on the BFOA algorithm [19]. With the aid of artificial bacteria, ICHB is completely capable of looking for greater residual energy nodes in the network and locating them. When it comes to initialization, ICHB is a fully distributive method that does not require centralized assistance (i.e., BS). Furthermore, the authors proposed the ICHB-HEED protocol, in which the ICHB algorithm was implemented on the HEED platform, which demonstrated efficient performance in the selection of better CHs (in terms of residual energy), the determination of an optimal and stable CH count per round, and the extension of the network lifetime when compared to the HEED protocol. When there is insufficient information, a fuzzy logic system (FLS) can create efficient results. FLS is a rule-based expert system that has the capability of producing efficient outcomes even when there is little information. In addition, FLS is very competent in creating real-time judgments by manipulating a semantic set of rules in order to deliver cutting-edge capabilities [20, 21].

A clustering technique for WSNs based on FLS employing battery level, node concentration, and distance factors was addressed by Gupta et al. [22] in their publication. Using FLS based on energy level and local distance characteristics, Kim et al. [23] reduced the overheads incurred during the CH selection operations in LEACH by a factor of two. Mao and Zhao [24] proposed a UCFIA protocol based on an uneven clustering technique using FLS and an enhanced ACO for intercluster routing operations, which was based on an unequal clustering procedure using FLS and an improved ACO for intercluster routing procedures. A type-2 FLS for clustering methods with better ACO has been addressed by Xie et al. [25], and this approach has been followed for intercluster communication in this paper. It was proposed by DUCF [26] to use FLS in the establishment of unequal-sized clusters in order to improve the network's load balancing capability.

Papers [27–32] discuss the various methods of the CH election process like CHs that are selected based on the amount of residual energy present, distance, and node density. Once a set of CHs has been selected, a message is broadcast by the CHs to the sensor devices in order to initiate cluster formation. At this point, when the sensor device receives messages from different CHs, it utilizes the intracluster communication cost to determine which CH is the best choice. This strategy aids in the correct load balancing of clusters among themselves. If any SN does not get this message, it automatically elects itself as a CH after each round in which it participates. The paper discusses an energy enhancement in LEACH using fuzzy logic called EE-LEACH [33–35]. This method prolongs the lifespan of WSNs and also performs load balancing using equal energy dissipation. The CH and cluster formation elections are conducted using rank-based fuzzy inference systems. However, this work does not consider the various other parameters in CH election and construction, such as average energy and number of neighbor's nodes. Papers [36–38] consider the IoT environment for enhancing the lifetime of heterogeneous networks. Moreover, the works presented are efficient and prolong the lifetime. Alshamrani [39] introduce a study of the IoT and artificial intelligence implementations for remote healthcare monitoring systems. This work categorizes the various things of the IoMT systems in the field of healthcare. Saba et al. [40] discuss a secure and energy-efficient framework using IoMT for e-healthcare systems. This work manages the data transmission in a secure manner but suffers from a large network overhead.

## 3. System Model

**Figure 1:**
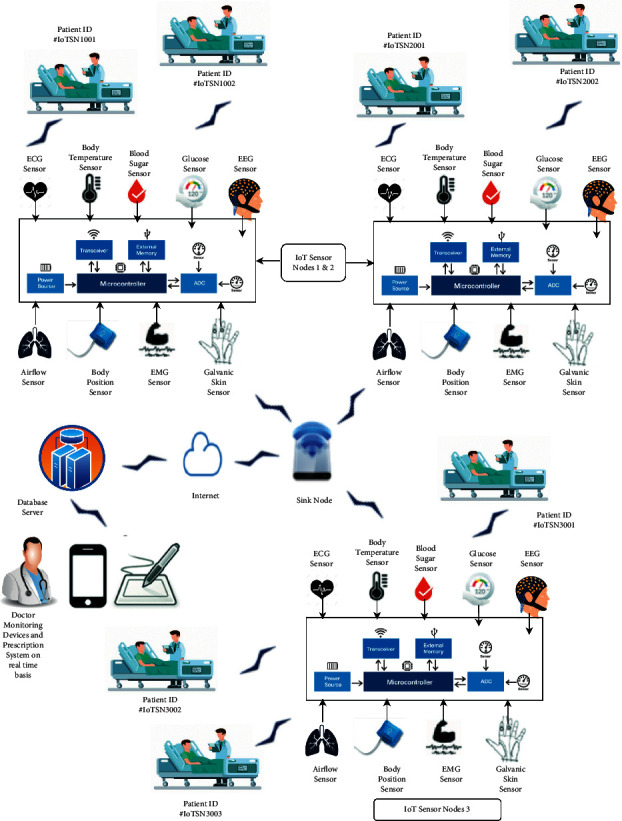
Scenario of the optimization of artificial intelligence system using IoT biosensors networking for healthcare problems.

In today's scenarios, biosensors-based IoT networking systems play a vital role in digital healthcare. In IoT networking systems biosensors are used to collect medical information and transmit the same using the wireless networks over the server or any web or mobile application. By using the abovementioned technology and drastic improvement in the healthcare systems, the healthcare systems can control patient treatment remotely over the Internet. These sensors collect the data and measure the human activities from physical to sleep level and mental to stress levels such as arterial pressure, oxygen level, blood alcohol level, glucose level, heart rate, and pulse. These sensors systems also alert the doctor if any health issue is detected during the measurement. Most of the devices collect data in a compassionate manner that is very useful for the treatment of the patient remotely on a real-time basis in case of various acute diseases. Thus, these systems can avoid the complication of the disease and treatment can be improved. A scenario is considered for the same and a detailed description is shown in Figure 1. Figure 1 shows a scenario of the optimization of an artificial intelligence system using IoT biosensors networking for healthcare problems where three IoT sensor nodes are deployed (for explanation point of view but in the real environment, 100 nodes are available). Each node has numerous sensors like ECG sensor, airflow sensor, body temperature, and position sensor, blood sugar sensor, glucose sensor, EEG sensor, EMG sensor, and galvanic skin sensor. These sensor nodes collect data from the patient and transfer the collected data to the database server with the help of sink nodes. Doctor monitoring devices are connected with the data server or control server. After getting the information doctor can suggest the prescription to the particular patient with the defined patient ID. Some of the significant assumptions are given as follows:

- Links are symmetric in nature and have equal capabilities for transferring data.
- Both homogeneous and heterogeneous sensor nodes are considered for deploying in the monitoring area evaluated in terms of their initial energy.
- Nodes are capable of transmitting and receiving data at the same time.
- Nodes are static after deployment but they can be relocated manually if required.

The homogeneous and heterogeneous sensor nodes are deployed in the monitoring area of the hospital healthcare systems. In the case of homogeneous networks, all the sensor nodes have the same amount of energy whereas three types of sensor nodes in terms of their energy heterogeneity are deployed in the case of heterogeneity networks. The *n* sensor nodes are deployed in the healthcare environment. Here, one and three levels of heterogeneity are considered for homogeneous and heterogeneous networks, respectively. One level of heterogeneity considers the only single type of node in terms of their battery power, i.e., *n*_1_ nodes [27, 41]. This network contains  *n* nodes with *E*_*o*_ initial energy nodes. Thus, the sum of energy of the network is *E*_Total_= *E*_*o*_*∗* *n*.

In the case of 3 levels of heterogeneity, *n*_3_, *n*_2_, and *n*_1_ nodes are deployed in the monitoring area of the healthcare environment. *E*_*n*_3__, *E*_*n*_2__, and *E*_*n*_1__ represent the energy of the number of *n*_3_, *n*_2_, and *n*_1_ nodes. The *n*_2_ and *n*_3_ nodes have Ψ and *ω* times higher energy than *n*_1_ nodes. Ψ and *ω* describe the energy fraction of *n*_3_ and *n*_2_ nodes, respectively. The *J* and *J*_*o*_ describe the proportion of *n*_3_ and *n*_2_ nodes, respectively. The number of different types of nodes is *n*_3_=*n∗J*, *n*_2_=*n∗* *J*_*o*_, and *n*_1_=*n∗*(1 − *J* −  *J*_*o*_). The energies of *E*_*n*_3__, *E*_*n*_2__, and *E*_*n*_1__ are *E*_*o*_*∗*(1+*ω*)*∗* *n*_*n*_3__, *E*_*o*_*∗*(1+Ψ )*∗n*_*n*_2__, and *E*_*o*_*∗n*_*n*_1__, respectively. The preliminary energy of *n*_3_ nodes is more by a factor of (1 + *ω*) and by a factor of (1 + *ψ*) for *n*_2_ nodes. The total energy of the network*E*_*Total*_ is *E*_*o*_*∗n∗*(1+Ψ*∗k*_*o*_+*k∗ω*).

The consumption of energy by the sensor nodes for data transmission over the short and long distance is given as follows [10, 11]:

(1) $$\begin{array}{c} E_{txs}=L\;*E_{elec}+L*E_{fs\;}*d^{2}\text{,}\;\text{if }d\leq d_{0},\\ E_{txl}=L\;*E_{elec}+L*E_{mp}*d^{4},\;\text{if }d>d_{0}. \end{array}$$

The consumption of energy by the nodes is for data receiving and data sensing is given as follows:

(2) $$\begin{array}{c} E_{rx}=\;E_{sx}=L*E_{elec}. \end{array}$$

where *E*_*elec*_, *E*_*fs*_, and *E*_*mp*_ are the consumption of energy in the electronic circuit, free, and multipath spaces, respectively, and *d*_0_ is threshold distance as given below:

(3) $$\begin{array}{c} d_{0}=\sqrt{\frac{E_{fs}}{E_{mp}}}. \end{array}$$

## 4. Proposed Methodology

In this section, an artificial intelligence-based algorithm is discussed, which effectively collects and transfers the data from the various deployed sensor nodes. The sensors (like ECG sensor, airflow sensor, body temperature and position sensor, blood sugar sensor, glucose sensor, EEG sensor, EMG sensor, and galvanic skin sensor) are deployed in the hospital scenario with the help of sensor nodes. The sensors collect the information and forward the collected data with the help of sinks to the control server where doctors are connected to the monitoring devices and after getting that information, doctors can suggest the prescription remotely on a real-time basis. The detailed description of the data collection and transfer is indicated in Figure 1. Here, a metaheuristic method called the tunicate swarm algorithm (TSA) is considered for selecting the effective routing path for transferring the data. The TSA metaheuristic method has high-speed and efficient capabilities of exploitation and exploration, and based on that feature it has a very high convergence rate. The complete process of the tunicate swarm algorithm (TSA) for the efficient routing is given as follows:

**Step 1 .**

Initially, set the population (*tuni*_pop_) of the tunicate.

**Step 2 .**

After initializing the tunicate population (*tuni*_pop_), initialize the parameters with the maximum number of iterations such as *α*, *β*, *γ*, *δ*, and *itr*_total_.

**Step 3 .**

Calculating the new search positions and avoiding the conflicts among the various tunicates, a vector considered as $\overset{⟶}{A}$ is considered as follows:

(4) $$\begin{array}{c} \alpha =\frac{\beta }{\delta }, \end{array}$$

where gravity force *β*=*a*_2_+*a*_3_ − *γ* and water flow advection *γ*=2  × *a*_1_, where *a*_1_, *a*_2_, *a*_3_ are the random number between the range of 0 and 1. *δ* is calculated as follows which is a social force during the search agents.

(5) $$\begin{array}{c} \delta =tuni_{\text{pop}}\left(\text{min}\right)+a_{1}\;\times \;tuni_{\text{pop}}\left(\text{max}\right)-tuni_{\text{pop}}\left(\text{min}\right), \end{array}$$

where *tuni*_pop_(max) and *tuni*_pop_(min) are the subordinates and initial speeds for social interaction and their values are considered as 4 and 1, respectively.

**Step 4 .**

Drive the fitness function (FitFun(*tuni*_pop_)) and calculate the fitness values of each search agent (*tuni*_pop_).

**Step 5 .**

Compute the random value between 0 and 1 for *a*_1_, *a*_2_, *a*_3_, and *r*_*rn*  *d*_ using random function.

**Step 6 .**

Determine the value of *α*, *β*, *γ*, and *δ*, as mentioned in Step 3. Also, calculate the movement in the direction of neighbor (Best_neigh_) as follows:

(6) $$\begin{array}{c} {\text{Best}}_{\text{neigh}}=abs\left(\text{FitFun}\left(tuni_{\text{pop}}\right)-r_{rn\;d}\times tuni_{\text{pop}}\left(x\right)\right). \end{array}$$

**Step 7 .**

The updated position of the swarm is calculated as follows and swarm behavior can be converged as the best search agent:

Set swarm=0.

(7) $$\begin{array}{c} \text{swarm}=\left\{\begin{array}{c} \text{Fit Fun}\left(tuni_{\text{pop}}\right)+\alpha \times {\text{Best}}_{\text{neigh}}\text{ if }r_{rn\;d}\leq 0.5,\;\\ \text{Fit Fun}\left(tuni_{\text{pop}}\right)-\alpha \times {\text{Best}}_{\text{neigh}}\text{if }r_{rn\;d}>0.5.\; \end{array}\right. \end{array}$$

**Step 8 .**

By using the above steps calculate the two best solutions of the tunicate swarm behavior and update the best search position of the search agents as follows:

(8) $$\begin{array}{c} tuni_{\text{pop}}\left(x\right)=\;\frac{\text{swarm}}{\left(2+a_{1}\right)}. \end{array}$$

**Step 9 .**

Then set swarm=0.

**Step 10 .**

After that update the parameters *α*, *β*, *γ*, and *δ*.

**Step 11 .**

Repeat Steps 8–10 for each value of *x*.

**Step 12 .**

Return the best obtained optimal solution in terms of fitness function value FitFun(*tuni*_pop_).

The complete process of the proposed work is divided into two parts called the network setup phase and the data collection and transmission phase. In the network setup phase, the various types of sensors such as ECG sensor, airflow sensor, body temperature, and position sensor, blood sugar sensor, glucose sensor, EEG sensor, EMG sensor, and galvanic skin sensor are deployed with the help of sensor nodes. A high capability-based sink node is also deployed which collects data from the various existing sensor nodes. The deployed nodes are homogeneous and heterogeneous in nature. After sensor nodes deployment, the process of routing will start in which first of all cluster heads (CH) election process will start. We use the tunicate swarm algorithms for calculating the fitness value using various parameters. The highest fitness value node will be the CH for the current round.

CH selection: Initially, the process of selection of CH is based on the LEACH [7] protocol which is the very first protocol in WSNs. This process is based on probability which helps in electing in the cluster heads. Additionally, a threshold value is calculated for checking the eligibility to become the cluster head. This probability and threshold values of nodes depend on the various parameters which are given as follows:

(i) *Networks Residual Energy* refers to the ratio of the sum of the energy of each node to the total energy of the networks.
  (9) $$\begin{array}{c} F_{1}=E_{res}=\frac{\sum_{i=1}^{n_{n}}E_{res}\left(i\right)}{E_{t}}, \end{array}$$
where *E*_*res*_ and *E*_*t*_ are the residual and total energy of the networks.
(ii) *Node density* refers to the number of nodes in the range of the cluster head. This factor also helps in the CH selection of the dense area where more nodes are deployed.
  (10) $$\begin{array}{c} F_{2}=n_{n\;D}=\frac{\sum_{i=1}^{n_{n}}D_{\left(n_{n}\left(i\right)-n_{nNS}\left(i\right)\right)}}{n_{n}}\times \frac{1}{D_{\left(n_{n}\left(i\right)-f\_\text{SNs}\right)}}, \end{array}$$
where *n*_*nD*_ is the node density, *f*_SNs is the farthest sensor node, *D*_(*n*_*n*_(*i*)−Sink)_ is the Euclidean distance from *i*th node and sink, *D*_avg(*n*_*n*_(*i*) − Sink)_ is the average distance at the center of *i*th node and sink, and *D*_(*n*_*n*_(*i*)−*f*_SNs)_ is the Euclidean distance from *i*th node and the farthest SNs.
(iii) *Average energy of node* (*E*_avg_) refers to the ratio of the sum of the energy of each node to the total number of nodes in the networks.
  (11) $$\begin{array}{c} F_{3}=E_{\text{avg}}=\frac{1}{n_{n}}\sum_{i=1}^{n_{n}}E_{i}. \end{array}$$
(iv) *Number of neighbors surrounded of a node (n*_*nNS*_*):* this parameter indicates the number of sensor nodes in the surrounding of other sensor nodes.
  (12) $$\begin{array}{c} F_{4}=n_{nNS}=\frac{\sum_{i=1,j=1}^{n_{n\;\left(CH\right)}}D_{n_{n}\left(i\right)-n_{n}\left(j\right)}}{n_{n}\left(CH\right)}, \end{array}$$
where *D*_*n*_*n*_(*i*)−*n*_*n*_(*j*)_ is the distance among the *i*th and *j*th nodes in the cluster and *n*_*n*_(*CH*) is the total number of SNs in the cluster.
(v) *Distance between sensor node and sink:* it refers to the distance between the sensor nodes and sink. It helps in reducing the communication distance.
  (13) $$\begin{array}{c} F_{5}=D_{SNs-sinks}=\frac{\sum_{i=1}^{n_{n}}D_{\left(n_{n}\left(i\right)-\text{Sink}\right)}}{D_{\text{avg}\left(f\_\text{SNs}-\text{Sink}\right)}}\times \frac{1}{\sum_{i=1}^{n_{n}}D_{\left(n_{n}\left(i\right)-\text{Sink}\right)}/n_{n}}. \end{array}$$

The final fitness function will be the integration of the above defined parameters. For optimizing the CH election process, the main aim is to maximize the fitness function *F* as follows:

(14) $$\begin{array}{c} F=a\times F_{1}+b\times F_{2}+c\times F_{3}+d\times F_{4}+e\times F_{5}, \end{array}$$

where *a*, *b*, *c*, *d* and *e* are the weights that are considered for electing the CH with the inequality *a*+*b*+*c*+*d*+*e*=1. These weights' system depends on the applications for that sensor network designed.

In the second phase of the algorithm, gathered data is collected by the sensor nodes and sent to the CH; thereafter CH forwarded the collected data to the sinks with the help of various other CHs or directly.

## 5. Simulation Results and Analysis

In this section, the performance of the proposed scenario is compared with the existing protocols. Figure 1 shows a scenario of optimizing an artificial intelligence system using IoT biosensors networking for healthcare problems where three IoT sensor nodes are deployed (for explanation point of view but in the real environment, 100 nodes are available). The sensor nodes are homogeneous and heterogeneous in nature in terms of their battery power. Each node has numerous sensors like ECG sensor, airflow sensor, body temperature, and position sensor, blood sugar sensor, glucose sensor, EEG sensor, EMG sensor, and galvanic skin sensor. These sensor nodes collect data from the patient and transfer the collected data to the database server with the help of sink nodes and doctor monitoring devices are connected with the data server or control server. After getting the information doctor can suggest the prescription to the particular patient with the defined patient ID. In this work, we are trying to improve the performance of the sensor networks in terms of stability period and longevity along with reducing the consumption power.

The performance of the proposed method is compared to GAOC [38] and OptiGACHS-StSS [38] protocols with different fitness functions depending on different fitness factors. These cutting-edge procedures GAOC [38] and OptiGACHS-StSS [38] protocols were chosen based on a comprehensive performance in the literature review. GA-based optimized clustering called GAOC is the existing work, and in this work, the clustering process is optimized using GA fitness function. Moreover, OptiGACHS-StSS protocol is the further extension of the GAOC. The same performance measures are used to compare proposed protocols to existing protocols. The following is a discussion of the aforementioned procedures with the various performance metrics. When comparing the performance of GAOC [38], OptiGACHS-StSS [38], and proposed protocol certain important performance indicators are used. The following are the points that will be covered.

- Period of Stability: This element is crucial to network stability since it guarantees the network's data is distributed reliably. The stability time is defined as the number of rounds covered until the first node of any type, such as advanced, intermediate, normal nodes, has depleted the whole supply of energy. It becomes an important performance parameter to evaluate for performance evaluation in some applications, where even a small loss of data can have a large magnitude of effects on the network's performance. Thus, the longer the stability period, the more reliable the recommended routing protocols in any network.
- Longevity of Networks: The network lifespan is critical for many applications in which information distribution is the consequence of continual monitoring. The number of cycles completed until all nodes run out of energy during the data transfer phase is known as network lifespan.
- The number of dead nodes in relation to the number of rounds: The performance of the networks can be measured by a factor that indicates the state of the number of dead nodes as rounds pass. The network performance is considered to be increased when a certain number of rounds are completed in comparison to the number of live nodes.
- Throughput: It is the number of data packets successfully transferred to the sink. This is a recurrent parameter for nurturing the quality of services (QoS) to ensure the network's resilience. The single evaluation of network endurance is insufficient for obtaining the best of the best. As a result, the network's performance and the QoS parameter improve network performance while also boosting the trustworthiness of the suggested routing scheme.
- The remaining energy of the network: The network's total energy eventually decreases due to the energy consumed by nodes while connecting with other nodes or with the sink as data transmission progresses. This measure aids in revealing the total energy status of nodes after each round.

This section describes the simulation environment used to simulate the GAOC [38] and OptiGACHS-StSS [38] protocols. The simulation programme MATLAB version 2016 is used for simulation, which runs on Windows 10 and has an Intel Core i7 CPU 540 processor running at 3.74 GHz and 8 GB of RAM. A network of 100 *m* × 100 *m* is simulated with 100 number of nodes, with 70 J energy homogeneous and heterogeneous nodes distributed at random. The *n*_1_ nodes have initial energy of 0.5 Joules, the fraction of *n*_3_ and *n*_2_ nodes is *ω* = 2,  *ψ* = 1, respectively, and the number of *n*_3_ and *n*_2_ nodes is *J*=0.1 and *J*_0_=0.2, respectively. The *E*_efs_, *E*_amp_, *E*_da_, and *d*_0_ are the amplification energy for *d*  ≤  *d*_0_, amplification energy for *d*  >  *d*_0_, data aggregation energy consumption, and threshold distance parameter which are 10pJ/bit/m^2^, 0.0013 pJ/bit/m^4^, 5nJ/bit/signal, and 87 m, respectively. The search agents, number of rounds, confidence interval, and data packet size are considered as 80, 1000, 95%, and 2000 bits, respectively. The analysis of homogeneous networks is given as follows.

### 5.1. Analysis of Stability Period

**Figure 2:**
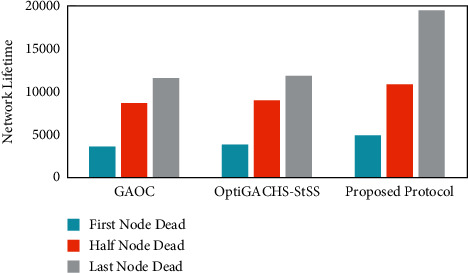
First, half and last node dead information for the GAOC [38], OptiGACHS-StSS [38], and proposed method.

As shown in Figure 2, the first node in the proposed protocol depletes its energy after 5176 rounds, but in OptiGACHS-StSS [38] and GAOC [38] protocols, the first node consumes its energy after 4035 and 3784 rounds, respectively. Compared to the OptiGACHS-StSS [38] and GAOC [38] protocols, the proposed protocol leads to a massive improvement in the stability period of 28.27 percent and 37.84 percent, respectively. Unlike the protocols OptiGACHS-StSS [38] and GAOC [38], which are considered for comparison, the proposed protocol ensures optimal energy-efficient CH selection, which improves the stability period. The addition of energy-efficient fitness factors in the fitness function formulation is a significant reason for the improvement in the stability period. Distance is taken into account. The use of an energy factor in the selection of CH helps to avoid uneven and sudden energy usage while also aiding in energy conservation. Furthermore, the node density factor guarantees that the nodes and sink have the shortest intracluster distance.

### 5.2. Network Lifetime

**Figure 3:**
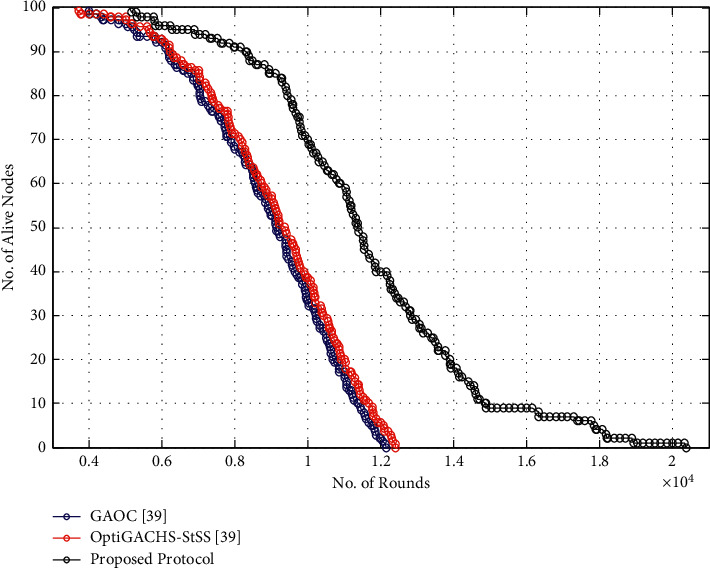
Number of alive nodes vs. number of rounds for homogeneous networks.

The proposed method covers 20429 cycles, whereas OptiGACHS-StSS [38] and GAOC [38] protocols cover 12426 and 12176 rounds, respectively, before total energy exhaustion of all nodes in the network, as shown in Figure 3. Compared to the OptiGACHS-StSS protocol, the proposed protocol has 8003 additional cycles, resulting in a 64.40 percent increase in network lifespan. Furthermore, compared to the GAOC [38] protocol, the proposed protocol improves network lifespan by 67.78 percent, respectively.

The energy-efficient fitness characteristics taken into account for the selection of CH are credited with this improvement. The node density factor lowers the communication cost of a cluster's sensor nodes. Because the node density factor promotes the CH selection of a node that is surrounded by more nearby nodes, this is the case. As a result, overall network energy is conserved, resulting in a more extended network lifetime. According to the primary data, the proposed protocol covers a more significant number of rounds at various phases of alive nodes. It is attributed to the optimal CH chosen under TSA operation having a lower energy consumption.

### 5.3. Network Remaining Energy

**Figure 4:**
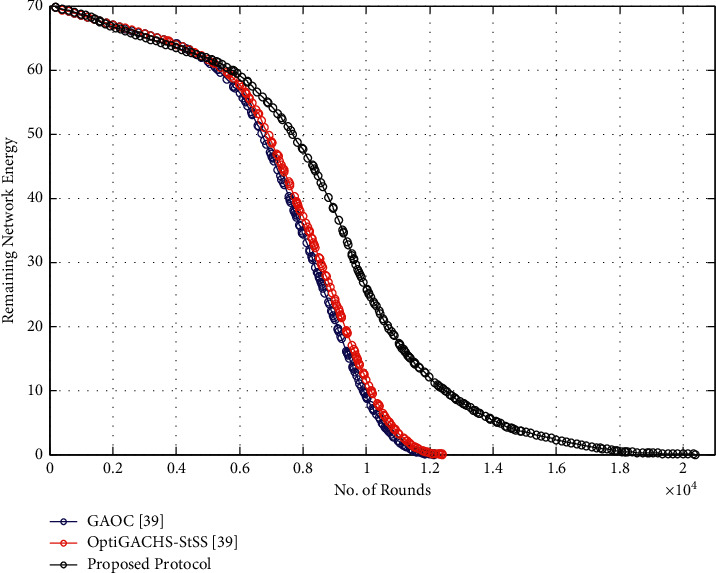
Remaining network energy vs. number of rounds for homogeneous networks.

This indicator measures the pace at which the network's energy is consumed. As data transmission progresses, the network's energy consumption decreases. Compared to OptiGACHS-StSS [38] and GAOC [38] protocols, the proposed protocol performs better in that it covers a higher number of cycles when data transmission is in progress, as seen in Figure 4. The proposed protocol covers the most rounds during network operation, whereas GAOC [38] covers the least amount of rounds. This is the case because energy-efficient CH selection costs a small amount of energy across all nodes. Furthermore, intracluster communication conserves node energy in the most effective way possible.

### 5.4. Number of CH per Round

**Figure 5:**
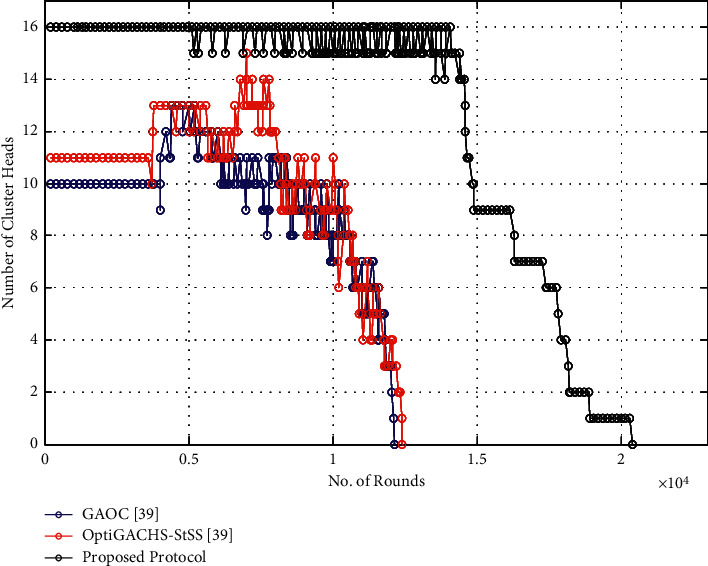
Number of cluster heads vs. number of rounds for homogeneous networks.

Figure 5 shows the number of cluster heads with respect to the number of rounds. The number of CHs varies up to 16 in the proposed method along with the OptiGACHS-StSS [38] and GAOC [38] procedures. The rate of generating the CH per round is more constrained in the proposed method, i.e., 15 to 16, whereas it is varied 10-12 in the case of OptiGACHS-StSS [38] and GAOC [38] procedures.

### 5.5. Throughput

**Figure 6:**
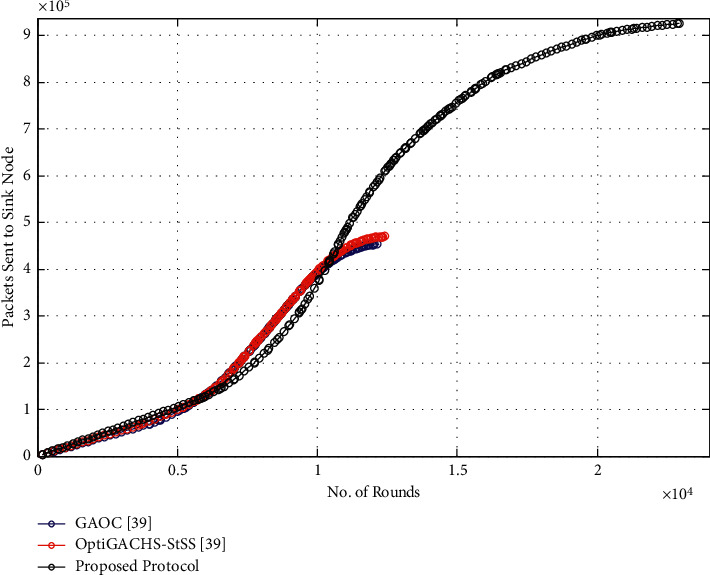
Number of packets sent to sink node vs. number of rounds for homogeneous networks.

As shown in Figure 6, the throughput of the proposed protocol is significantly increased, as it successfully sends 9.1 × 10^5^ data packets, whereas GAOC [38], respectively, transmits 4.4 × 10^5^ and 4.5 × 10^5^ data packets. The proposed protocol enhances throughput by 98.24 percent and 102.67 percent, respectively, compared to OptiGACHS-StSS [38] and GAOC [38] procedures, as seen in the throughput comparison study. This improvement can be attributed to the network's optimal CH selection, which also aids in network lifetime. As a result, nodes send data packets for extended periods, dramatically increasing throughput.

**Figure 7:**
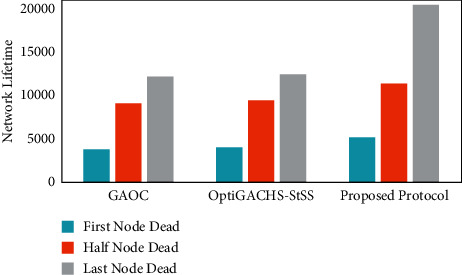
First, half and last node dead information for the GAOC [38], OptiGACHS-StSS [38], and proposed method.

Analysis of the heterogeneous networks is given as follows: As shown in Figure 7, the first node in the proposed protocol depletes its energy after 7188 rounds, but in OptiGACHS-StSS [38] and GAOC [38] protocols, the first node consumes its energy after 6607 and 6107 rounds, respectively. Compared to the OptiGACHS-StSS [38] and GAOC [38] protocols, the proposed protocol leads to a massive improvement in the stability period of 8.79 percent and 17.70 percent, respectively. Unlike the protocols OptiGACHS-StSS [38] and GAOC [38], which are considered for comparison, the proposed protocol ensures optimal energy-efficient CH selection, which improves the stability period. The addition of energy-efficient fitness factors in the fitness function formulation is primary in enhancing the stability period. Distance is taken into account. Using an energy factor in the selection of CH helps avoid uneven and sudden energy usage while also aiding in energy conservation. Furthermore, the node density factor guarantees that the nodes and sink have the shortest intracluster distance.

### 5.6. Network Lifetime

**Figure 8:**
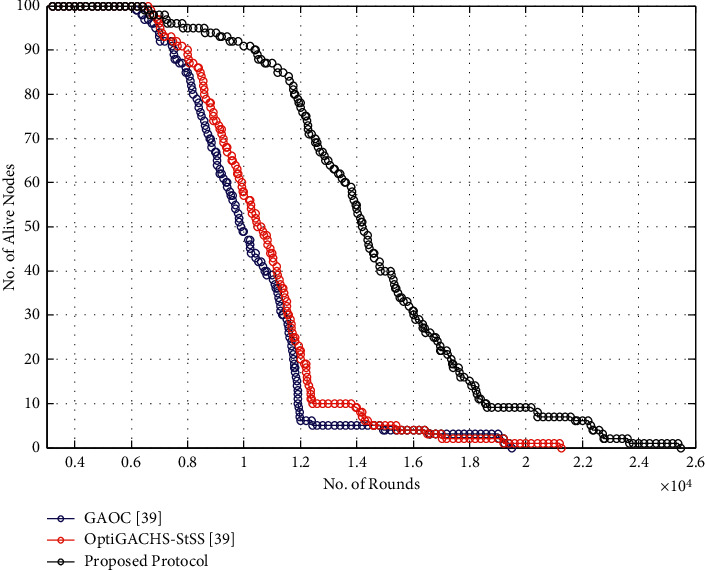
Number of alive nodes vs. number of rounds for heterogeneous networks.

The proposed method covers 25572 cycles, whereas OptiGACHS-StSS [38] and GAOC [38] protocols cover 21311 and 19487 rounds, respectively, before total energy exhaustion of all nodes in the network, as shown in Figure 8.

Compared to the OptiGACHS-StSS protocol, the proposed protocol has 4261 additional cycles, resulting in a 19.99 percent increase in network lifespan. Furthermore, compared to the GAOC [38] protocol, the proposed protocol improves network lifespan by 31.22 percent, respectively. The energy-efficient fitness characteristics taken into account for the selection of CH are credited with this improvement. The node density factor lowers the communication cost of a cluster's sensor nodes. Because the node density factor promotes the CH selection of a node that is surrounded by more nearby nodes, this is the case. As a result, overall network energy is conserved, resulting in a more extended network lifetime.

### 5.7. Network Remaining Energy

**Figure 9:**
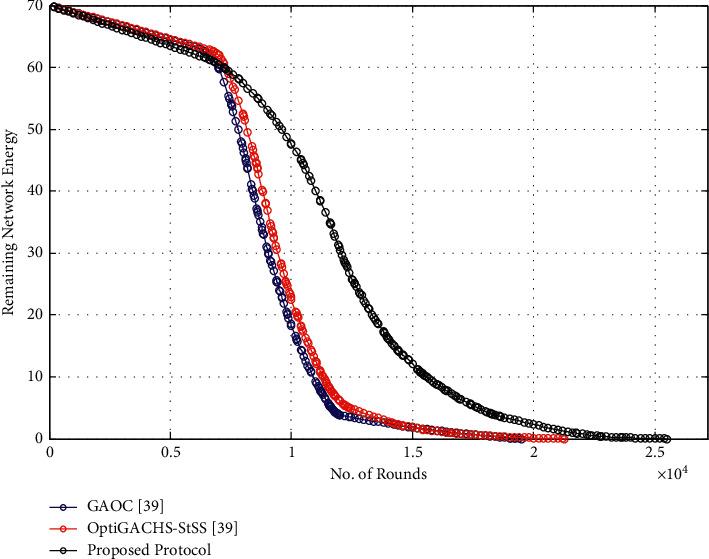
Remaining network energy vs. number of rounds for heterogeneous networks.

This indicator measures the pace at which the network's energy is consumed. As data transmission progresses, the network's energy consumption decreases. Compared to OptiGACHS-StSS [38] and GAOC [38] protocols, the proposed protocol performs better in that it covers a higher number of cycles when data transmission is in progress, as seen in Figure 9. The proposed protocol covers the most rounds during network operation, whereas GAOC [38] covers the least amount of rounds. This is the case because energy-efficient CH selection costs a small amount of energy across all nodes. Furthermore, intracluster communication conserves node energy in the most effective way possible.

### 5.8. Number of CH per Round

**Figure 10:**
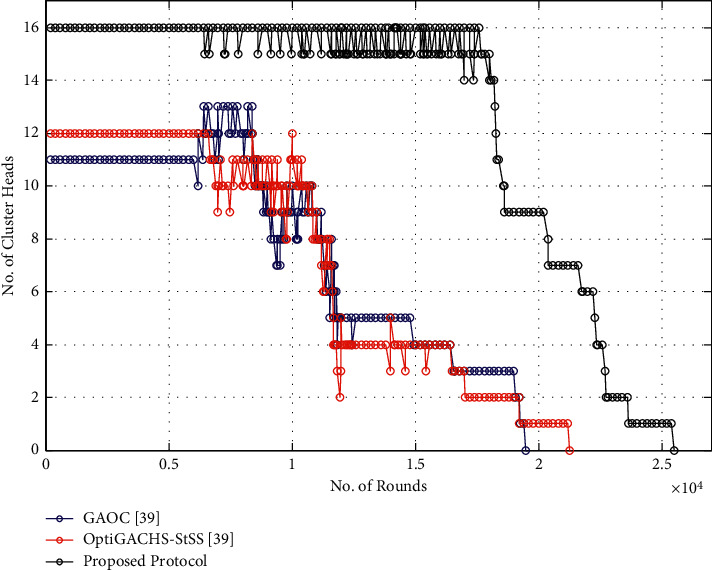
Number of cluster heads vs. number of rounds for heterogeneous networks.

Figure 10 shows the number of cluster heads with respect to the number of rounds. The number of CHs varies up to 16 in the proposed method along with the OptiGACHS-StSS [38] and GAOC [38] procedures. The rate of generating the CH per rounds is more constrained in the proposed method, i.e., 15 to 16 whereas it is varied 10-12 in the case of OptiGACHS-StSS [38] and GAOC [38] procedures.

### 5.9. Throughput

**Figure 11:**
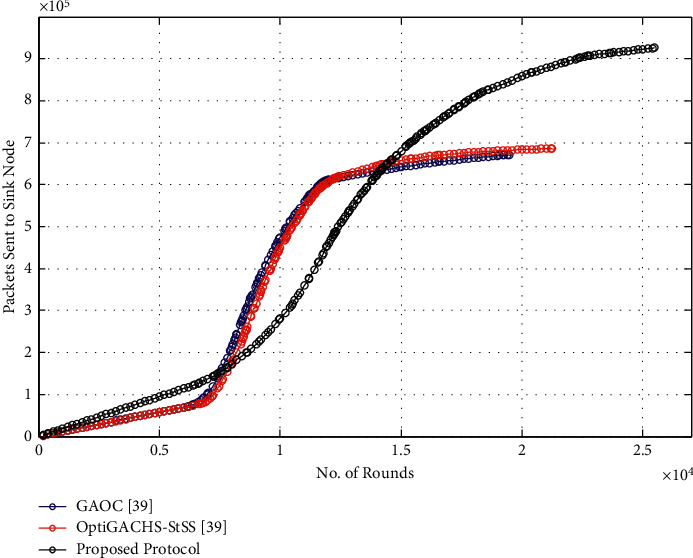
Number of packets sent to sink node vs. number of rounds for heterogeneous networks.

As shown in Figure 11, the throughput of the proposed protocol is significantly increased, as it successfully sends 9.2 × 10^5^ data packets, whereas GAOC [38], respectively, transmits 6.8 × 10^5^ and 6.9 × 10^5^ data packets. The proposed protocol enhances throughput by 35.29 percent and 33.33 percent, respectively, compared to OptiGACHS-StSS [38] and GAOC [38] procedures, as seen in the throughput comparison study. This improvement can be attributed to the network's optimal CH selection, which also aids in network lifetime. As a result, nodes send data packets for extended periods, dramatically increasing throughput.

## 6. Conclusion

This paper proposes an optimized artificial intelligence system using IoT biosensors networking for healthcare problems, efficiently collecting and transmitting the data from the deployed sensor nodes. This work has discussed an optimized tunicate swarm algorithm with the fitness parameters such as distance, proximity, number of neighbors surrounded of a node, and various energies. The proposed protocol leads to network stability compared to OptiGACHS-StSS [38] and GAOC [38] protocols to a massive improvement in stability period of 28.27 percent and 37.84 percent, respectively. There are many reasons for such modification, which are as follows: first, the addition of energy-efficient fitness factors like distance, energy, and proximity in the fitness function formulation. Secondly, using an energy factor in the selection of CH helps avoid uneven and sudden energy usage while also aiding in energy conservation. Furthermore, intracluster communication conserves node energy in the most effective way possible.

## Data Availability

Data will be available on request to the submitting author (yashveersingh85@gmail.com), if required.
